# Great Expectations: Induced pluripotent stem cell technologies in neurodevelopmental impairments

**DOI:** 10.7150/ijms.51842

**Published:** 2021-01-01

**Authors:** Xue Zhang, Zilong Li, Yi Liu, Zhongtao Gai

**Affiliations:** 1 Pediatric Research Institute, Qilu Children's Hospital, Cheeloo College of Medicine, Shandong University, Ji'nan 250022, China.; 2 Jinan Pediatric Research Institute, Jinan Children's Hospital, Ji'nan 250022, China.; 3 Neonatal Intensive Care Unit, Children's Medical Center, The Second Hospital of Shandong University, Ji'nan 250033, China.

**Keywords:** iPSCs, IPSCS Disease Models, Neurodevelopmental Impairments, Autism Spectrum Disease, Pediatric Epilepsy, Down Syndrome, Organoid

## Abstract

Somatic cells such as skin fibroblasts, umbilical cord blood, peripheral blood, urinary epithelial cells, etc., are transformed into induced pluripotent stem cells (iPSCs) by reprogramming technology, a milestone in the stem-cell research field. IPSCs are similar to embryonic stem cells (ESCs), exhibiting the potential to differentiate into various somatic cells. Still, the former avoid problems of immune rejection and medical ethics in the study of ESCs and clinical trials.

Neurodevelopmental disorders are chronic developmental brain dysfunctions that affect cognition, exercise, social adaptability, behavior, etc. Due to various inherited or acquired causes, they seriously affect the physical and psychological health of infants and children. These include generalized stunting / mental disability (GDD/ID), Epilepsy, autism spectrum disease (ASD), and attention deficit hyperactivity disorder (ADHD). Most neurodevelopmental disorders are challenging to cure. Establishing a neurodevelopmental disorder system model is essential for researching and treating neurodevelopmental disorders. At this stage, the scarcity of samples is a bigger problem for studying neurological diseases based on the donor, ethics, etc.

Some iPSCs are reprogrammed from somatic cells that carry disease-causing mutations. They differentiate into nerve cells by induction, which has the original characteristics of diseases. Disease-specific iPSCs are used to study the mechanism and pathogenesis of neurodevelopmental disorders. The process provided samples and the impetus for developing drugs and developing treatment plans for neurodevelopmental disorders. Here, this article mainly introduced the development of iPSCs, the currently established iPSCs disease models, and artificial organoids related to neurodevelopmental impairments. This technology will promote our understanding of neurodevelopmental impairments and bring great expectations to children with neurological disorders.

## Introduction

Stem cells are the source of various tissue cells in the human body and can self-replicate, undergo proliferation, and multi-directional differentiation. Since the end of the 20^th^ century, embryonic stem cells (ESCs) have become the focus of research worldwide. However, ESCs are often involved in ethical and moral issues due to different customs and cultural backgrounds in different countries. At the same time, the acquisition and preservation of ESCs are also limited by practical conditions. Also, immune rejection also affects its clinical application. Therefore, using non-embryonic materials to generate induced pluripotent stem cells (iPSCs) directly has become a new direction in the development of stem cell research. After being reprogrammed, stem cells with pluripotency are transformed from differentiated somatic cells called iPSCs. IPSCs are similar to ESCs, showing self-renewal and pluripotency. In past decades, iPSCs were one of the most exciting discoveries. In 2006, mouse fibroblasts were reprogramed with four transcription factors (OCT4, SOX2, KLF4, and cMYC) by Shinya Yamanaka, namely IPSCs, which were characterized by ESCs 1. In 2007, Takahashi successfully obtained human induced pluripotent stem cells (hiPSCs) by human fibroblasts 2. In the same year, Yu and Thomson also reported that different transcription factors (POU5F1, Sox2, NANOG, and Lin28) induced hiPSCs 3. Since then, many researchers have also adopted the same method to obtain iPSCs 4-9. In 2009, Chinese scientists 10 firstly reported that iPSCs were used to obtain viable and reproductive mice by tetraploid blastocyst injection, thus proving the pluripotency of iPSCs for the first time in the world. Similar to human embryonic stem cells (hESCs), hiPSCs can differentiate into most types of cells 11-15. Additionally, there is no moral dispute, and tissue samples are easy to be obtained (such as peripheral blood, urine, skin, etc.) 16. The more valuable is that hiPSCs are reprogrammed from patients' cells to avoid immune rejection 17. For more than ten years, researches have grown explosively in this area. IPSCs have been widely engaged in various fields, such as disease modeling, drug discovery, and regenerative medicine 18, 19. Great progress has been made in some diseases such as geriatrics, tumors, retinopathy 20-23.

Neurodevelopmental disorders are a broad group of chronic developmental brain dysfunctions, which are primarily due to diseases caused by various hereditary or acquired etiologies affecting the brain's multipotent area, including cognition, exercise, social adaptability, and behavior 24 principally including comprehensive sexual retardation/mental disability, autism spectrum disease, Epilepsy, and attention deficit hyperactivity disorder 25.

It is hard to cure most neurodevelopmental disorders (NDDs). So far, the pathogenesis of NDDs is not clear but no effective treatment method has been shown. Stem cell transplantation treatment and drug intervention therapy only play a role in relieving the patient's disease. Therefore, the establishment of NDD model systems is crucial for the research and treatment of neurodevelopmental disorders. At this stage, the scarcity of samples is a bigger problem for studying neurological diseases. The establishment of a perfect neuronal disease model is the primary goal of studying neurological diseases. The purpose of studying neurological diseases is to determine the mechanism that causes diseases and determine therapies to improve cell damage. A good neurological disease model is one of the prerequisites for accomplishing the goal. In view of the fact that children's nerve cells are rarely sampled for neuropathological research during the pathogenesis, the current understanding of human neurological diseases is mainly based on post-mortem analysis of children with advanced disease. Although transgenic mouse models provide simulations of human neurodevelopmental disorders, this method is limited to single-gene diseases, so it only represents a few diseases. Furthermore, species differences are a major problem with this technology. The emergence of hiPSCs will hopefully solve this problem. Compared with animal models, disease models based on the cultivation of hiPSCs have outstanding advantages. Obtained easily, these cells can differentiate into any desired cells theoretically, and are applied to the study of any target cells, which are not affected by species-specific differences. The iPSCs disease models have been applied to some organ systems 21-23, 26-32. Besides, the current reprogrammed iPSCs have been widely used to study disease models and cell replacement therapy in animal experiments, which is possible to study the pathogenesis of diseases 33.

Generally, it is not suitable to study central nervous system diseases (CNSDs) *in vitro*, and it is difficult to obtain human nerve tissues. Therefore, patient-derived iPSCs provide a unique opportunity to study diseases of the central nervous system. Under the action of specific nerve transcription factors, hiPSCs differentiate into neurons, including spinal cord motor neurons, dopaminergic neurons, neural crest cells, etc. 34, 35. With the differentiation of iPSCs into nerve cells, a good neurological disease model is established. For some genetic diseases, the iPSCs from these patients carry genetic mutations pattern of diseases and have similar pathological/phenotypes changes 36. Using iPSCs, researchers studied the mechanism of neurological diseases, developed high-throughput screening (HTS) of drugs, and tested drug toxicology 37-39. Drug experiments are conducted directly on nerve cells induced from iPSCs so that further personalized medicine is available.

Here, this article summarized the research progress of iPSCs in various pediatric neurodevelopmental impairments, including autism spectrum disease (ASD), Epilepsy, Down syndrome, and rare diseases related to neurodevelopmental disorders, focused on modeling and mechanism research, discussed the current challenges and opportunities in iPSCs, and provided guidance and direction for the research of pediatric neurodevelopmental impairments.

## IPSCs induction strategies

With iPSCs technology development, there have been various methods for transmitting reprogramming transcription factors into cells to produce iPSCs. The researchers have been working hard to optimize the delivery method of reprogramming factors. Reprogramming is reversing the differentiation and proliferation of cells and restoring them to totipotency by epigenetic modification without changing the gene sequence, which is a complex process. In addition to being regulated by intracellular factors, reprogramming is also regulated by extracellular signaling pathways 40, 41. Reprogramming of iPSCs derived from somatic cells is achieved by forced expression of the transcription factors: Oct4, Klf4, Sox2, and c-Myc. These factors, combined with environmental factors, create a stable internal potential network that allows iPSCs to have unlimited self-renewal capabilities. According to different vectors, the current methods for reprogramming are roughly divided into integrated and non-integrated methods. Integrated reprogramming often uses vectors such as retrovirus and lentivirus to carry out gene introduction and integration, and finally, realize reprogramming. Because the general retrovirus infection of cells depends on the state of cell division, the range of cells used for reprogramming by non-retro-dependent lentiviruses that infect both dividing and non-dividing cells is wider 3. Although the method of genome integration of viral vectors provides the initial technology for the generation of iPSCs, its random integration phenomenon may lead to insertional mutations, interfere with the regulation of endogenous gene expression, or be abnormally reactivated in terminally differentiated cells. On the other hand, researchers have successively developed several non-gene integrated reprogramming methods. Non-integrative reprogramming reduces the changes to chromosome structure, and to a certain extent, reduces the possibility of gene mutation and tumorigenesis. Sendai virus, the classic non-integrating vectors 42, and adenovirus 43 can mediate the transient expression of transcription factor genes in host cells, induce pluripotency of cells, and induce iPSCs without virus integration 44. IPSCs were obtained with PiggyBal transposon transposition instead of virus integration 45. Subsequently, the fibroblasts were successfully reprogrammed into iPSCs through plasmid 46 or plasmid transfection 47, 48 carrying transcription factors. Besides, methods such as the non-integrating reprogramming factors were widely used. Oct4, Sox2, Klf4, C-MYC, Nanog, lin28, Nr5a2, Micro RNA302/367, and positive/negative screening marker genes were constructed into a non-integrated plasmid. The iPSCs without non-integrated plasmids were screened to ensure that no exogenous genes were inserted 49. Although the vector reprograms somatic cells to obtain iPSCs, the reprogramming efficiency using these vectors is relatively low. In recent years, many experimental methods were used to reduce foreign genes' random integration to improve safety. At present, mouse and human fibroblasts were successfully cultured into iPSCs utilizing modified RNAs 50, 51, recombinant proteins 52, and small molecule compounds 53-55. In 2014, mature double-stranded RNAs (miRNAs) were used to reprogram mouse and human cells into iPSCs 50. This reprogramming method does not require vector-based gene transfer, so it expresses great potential in biomedical research and regenerative medicine. On the other hand, recent studies have shown no DNA manipulation by directly reprogramming proteins to target cells in mice or humans. This reprogramming method could be used to generate iPSCs, and no foreign DNA was integrated into the host genome. 52. Recently, it is worth noting that some researchers screened 10,000 small molecule compounds. FSK2-Me-5HT and D4476 were selected as replacements for the reprogramming factor Oct4. Mouse iPSCs could be obtained by adding only seven small molecule compounds (VC6TFZ, VPA, E616452, CHIR, D4476 FSK, 2-Me-5HT) to the medium, and the efficiency of reprogramming into iPSCs was 0.2% 53. The small molecule-induced iPSCs were similar to ESCs in terms of gene expression profile, epigenetic status, potential differentiation ability, and germline transmission. This demonstrated that small molecule compounds could also be employed to reprogram somatic cells to a pluripotent state 53. It suggested the possibility of generating hiPSCs for clinical applications without complicated operations. At present, the latest advances in reprogramming technology are focusing on developing a new method that does not require reprogramming factors to enter cells to achieve the successful transformation of iPSCs. For example, some researchers have discovered some antibodies that stimulated intracellular pathways, which eventually activated target genes like reprogramming factors 56. These antibodies acting on the cell surface replaced transcription factors used in reprogramming. It prevented genes with potential carcinogenic risks such as c-Myc from entering the cell and eliminated the random integration of foreign genetic material into the genome to destroy functional genes or activate oncogenes. However, there were also many technical problems with reprogramming technologies, such as clonal variability, variable differentiation across replicates, and the low throughput manner of this technique. These problems are expected to be resolved with the rapid development of molecular biology and cell biology.

The cell adhesion matrix's mechanical properties and micro/nanostructure may also play a key role in cell reprogramming. Researchers have found that biophysical cues, which were called parallel microgrooves on the surface of cell-adhesive substrates, could replace the effects of small-molecule epigenetic modifiers (VPA and TCP) and enhance the ability of cells to reprogram to iPSCs 57. The specific mechanism depended on the mechanomodulation of the cells' epigenetic state regulation. The micro-grooved surface reduced histone deacetylase activity and up-regulated the expression of WD repeat domain 5 (WDR5) (a subunit of H3 methyltransferase), resulting in increased histone H3 acetylation and methylation 57. Also, one team coated stable nanoparticles on Petri dishes. Then they found that nanoparticles contributed to maintaining the pluripotency of iPSCs and promote the proliferation of iPSCs without feeder cell co-culture. 58. Some researchers have also compared Petri dishes with topological patterns on the surface of different sizes or Petri dishes with a smooth surface. They found that a culture surface with a small topological feature size (1.6 μm) promoted the differentiation of iPSCs to the ectoderm, while a large topological feature size (8 μm) culture surface inhibited cell self-renewal 59. The cultivation of stable induced pluripotent stem cells in the absence of exogenous conditions is essential for producing clinical-grade therapeutic cells. Current studies have found that the pluripotency of mouse and human stem cells was regulated by matrix stiffness. For example, hydrogels' design and the surface density of cell-binding domains can promote the proliferation of human embryonic stem cells and iPSCs under long-term exogenous culture conditions. It is important to maintain the pluripotency of these cells 60. According to reports, the mouse embryonic fibroblasts transduced with OSKM factor were cultured on a softer hydrogel (100 Pa). Compared with a harder hydrogel (≥1 kPa), the reprogramming efficiency has been tripled. Soft matrix promoted cell reprogramming by activating epithelial-mesenchymal transformation (EMT) and enhancing the expression of pluripotency markers 61. Similarly, the mouse embryonic fibroblasts were embedded in three-dimensional hydrogels with different mechanical properties for culture, and it was found that hydrogels with less rigidity provided higher reprogramming efficiency 62. These research results presented important significance for the optimization of biomaterials for iPSCs induction.

## Application of iPSCs in ASD

ASD is a complex, lifelong neurodevelopmental disorder that often begins before the age of 3 years. The etiology of ASD is complex, with specific clinical phenotypes and genetic heterogeneity. Patients often have clinical manifestations, such as language communication difficulties, social behavior disorders, and repetitive stereotypes 63. ASD is defined genetically as a syndromic or idiopathic case. Syndromic cases are caused by a known genetic disorder, including Rett syndrome (MECP2 gene mutation), Fragile X syndrome, Timothy syndrome, etc. 64-66. In contrast, idiopathic cases have an unknown genetic cause. It is not clear about the pathological mechanism of ASD, but existing studies have shown that its pathogenesis is closely related to genetic factors 67. Due to the scarcity of human nerve samples, current research mainly relies on animal and cell models. However, the genetic background of ASD is heterogeneous, and it is difficult for transgenic animal models to completely simulate each ASD disease's occurrence. IPSCs derived from patients' somatic cells differentiate into adult neurons or other nerve cells *in vitro*, with the same genetic background as patients, which makes them an ideal model for studying ASD's pathogenesis ASD 68, 69. In fact, in some ASD research, iPSCs technology has been employed to model ASD and explore its underlying mechanisms.

Rett syndrome (RTT) is an x-linked single gene disease that seriously affects children's psychomotor development 70. The clinical cases are mostly girls. Non-random inactivation of the X chromosome may play a role in the genetic process of the disease. Currently, the MECP2 gene is closely related to its pathogenesis, while the CDKL5 gene appears in atypical RTT 70. In 2010, a research team 71 successfully induced and differentiated iPSCs from children with MECP-2 gene mutation RTT for the first time. With the animal model, a study further verified that the RTT-iPS derived from MECP-2 gene mutation provided a cell model for people to study further the pathogenesis of RTT and drug screening 72. On the other hand, Amenduni et al. 73 generated iPSCs derived by fibroblasts from CDKL5 gene mutant RTT children. These iPSCs further differentiated into neural cells used to study the function of the CDKL5 gene and the mechanism of non-random inactivation of the X chromosome. In 2011, Aaron Y.L et al. 74 successfully constructed the Rett syndrome disease development model utilizing hiPSCs. They found that mutant RTT-hiPS cell-derived neuron cell bodies became smaller compared to the isogenic control hiPS cell-derived neurons. Also, other studies utilized RTT patient-derived iPSCs to generate differentiated RTT cortical neurons. Compared to normal cortical neurons, these RTT cortical neurons had fewer glutamatergic synapses, reduced dendritic arborization, smaller somas, and fewer dendritic spines, which were defined as synaptic deficits 66, 75-77. After, some researchers generated iPSCs from patients with the MECP2 duplication syndrome (MECP2dup), carrying different duplication sizes. The result showed that cortical neurons derived from these different MECP2dup iPSCs lines increased synaptogenesis and dendritic complexity 78. Other functional defects were also detected in neurons derived from iPSCs in RTT patients, including altered calcium signaling and electrophysiological defects 71, 76, 79. As well as, astrocytes play a role in neuronal support in the formation and maintenance of synapses and the reuptake of neurotransmitters in synapses. Increased astrocyte formation in RTT cultures suggested that glial MECP2 might play a role in regulating neuronal maturation and dendritic dendrites. In contrast, neuronal networks' complexity in mature RTT cultures was reduced 71, 76, 79. It was also reported that microtubule (MT)-dependent vesicle transport was altered in Mecp2-deficient astrocytes derived from hiPSCs. Importantly, a brain-penetrant MT-stabilizing natural product, named administration of Epothilone D, was found to restore MT dynamics in astrocytes derived from Mecp2-deficient iPSCs *in vitro*. Finally, they reported that relatively low doses of Epothilone D partially reversed the impaired exploratory behavior in Mecp2 deficient male mice 80. More interestingly, some researchers found that human neurons differentiated from iPSCs induced from Rett syndrome had significant defects in KCC2 expression. This defect delayed the functional conversion of GABA from excitement to inhibition. In Mecp2 deficient neurons, overexpression of KCC2 improved GABA function defects, which indicated that KCC2 played an important role in Rett syndrome. Their research suggested that restoring the KCC2 function in Rett neurons might be a potential treatment for Rett syndrome 81. And these models represented a promising cellular tool to facilitate therapeutic drug screening for ASD.

Fragile X syndrome (FXS) and Timothy's syndrome also often present the clinical signs of ASD. Patients are often accompanied by moderate to severe intellectual disability, language delay, growth, movement disorders, hyperactivity, and anxiety 25, 82, 83. FXS is the result of reduced FMR1 gene expression. The FMR1 gene encodes FMRP, which is an RNA binding protein inhibiting mRNA translation 25. The regulation of mRNA translation is important for synaptic plasticity and neuronal maturation, making this gene essential for normal brain development. Timothy Syndrome is caused by a mutation in the CACNA1C gene 25 encoding an L-type voltage-gated calcium channel (LTCs). LTCs play an important role in neuronal development by promoting dendritic growth and differentiation. A point mutation in the gene encoding CaV1.2 leads to Timothy syndrome 84. A study has found that hiPSCs-derived Timothy syndrome neurons displayed impaired calcium signaling and electrophysiology and presented defects in activity-dependent transcription 83. Another researcher found that channels with the Timothy syndrome alteration cause activity-dependent dendrite retraction in rat and mouse neurons and induced pluripotent stem cell (iPSCs)-derived neurons from individuals with Timothy syndrome 84. The dendritic contraction was not related to the penetration of calcium through the mutant channel, but was related to the ectopic activation of RhoA and was inhibited by channel-related overexpression GTPase Gem. These results indicated that CaV1.2 activated RhoA signaling independent of Ca^2+^. Their data revealed a direct cellular connection between the mutations that caused Timothy syndrome in LTCs and defects in dendritic remodeling. They showed that iPSCs-derived neurons reproduced the neuronal defects observed *in vivo* 84. Some studies created hiPSCs models of FXS and Timothy Syndrome to study their neurons' phenotype 83, 85, 86. One group found that iPSCs-derived forebrain neurons from FXS individuals showed defects in initial neurite outgrowth 85. FXS forebrain neurons were differentiated from these iPSCs and displayed defective neurite initiation and extension 85. Research data showed that abnormal regulation of neural differentiation and axon guidance genes were regulated by RE-1 silent transcription factor (REST) in neurons derived from FXS. Because FMRP is involved in the microRNA (miRNA) pathway, they employed miRNA array analysis and discovered several miRNAs' dysregulations in FXS-derived neurons. This research connected FMRP and REST through the miRNA pathway, providing a new direction for the development of FXS 87. Interestingly, some researchers engaged a CRISPR/Cas genomic engineering technique to ablate CGG repeats in FXS patient-derived hiPSCs 83. It was restored the expression of FMR1 mRNA and FMRP protein. It's a pity that their study did not assess any phenotypic reversal. HiPSCs derived from these patients had an abnormal tendency to differentiate. Differentiated neurons had reduced expression of genes marking the lower cortex 83.

Genetically, most cases of ASD are idiopathic or unexplained. Symptomatic ASD cases are caused by known genetic diseases, such as Rett or Fragile X syndrome. In contrast, idiopathic ASD cases are not yet known to be inherited. Therefore, it is particularly important to use iPSCs as a research measure for these cases to study the pathogenesis. In recent years, some achievements have been made in establishing organoid-3D brain-like structures to study ASD. One group has reported that they utilized hiPSCs-derived organoids to model ASD 25. This was the first published model of non-syndromic and idiopathic autism independent of any other disorders and made a phenotypic assessment 25. Researchers have used three-dimensional neural cultures (organoids) from iPSCs to study neurodevelopmental changes in patients with severe idiopathic ASD 88. Transcriptome and gene network analysis showed that genes involved in cell proliferation, neuronal differentiation, and synapse assembly were up-regulated. ASD-derived organoids showed accelerated cell cycle and excessive production of GABAergic inhibitory neurons. Through RNA interference, they found that overexpression of the transcription factor FOXG1 led to the overproduction of GABAergic neurons. Moreover, Gene network modules and FOXG1 expression changes were positively correlated with the severity of symptoms. Their data indicated that the shift in GABAergic neurons' fate caused by FOXG1 was a developmental precursor of ASD 88. Besides, morphological analysis of neuron maturation and synapse formation showed that neurons' density in autistic organs increased, and the total number of synapses increased 88. Shcheglovitov and colleagues 89 made use of hiPSCs to construct functional neurons to study ASD and found that the expression of shank3 protein in shank3 mutant neurons was reduced. Their synapses were significantly reduced, making the synaptic connection defective. In 2019, it was reported that the dendrite length, complexity, and the number of iPSCs-derived cortical neurons from ASD individuals with the gene SHANK2 mutation increased, and it was demonstrated that ASD neuron defects were not just reduced in function 90. Utilizing brain organoids derived from these cell lines, RNASeq technology was used to identify differential expression of genes in pathways related to nervous system development, neurogenesis, neuronal differentiation, forebrain development, axon guidance, and WNT-βcatenin signaling 91. These results showed that the iPSCs models were employed to explore the complex pathological mechanism of autism and provided a new platform for seeking treatment methods for autism.

## Application of iPSCs in Down Syndrome (DS)

Down Syndrome (DS) is a complex chromosomal disorder that causes multiple structural and functional derangements of neural systems throughout all life stages that cause multiple structural and functional derangements of neural systems throughout all life stages and impacts overall neurodevelopment 92, 93. Primary neuronal cells and tissues are beneficial, but limited both in supply and experimental manipulability. To better understand the cellular, molecular, and pathological mechanisms involved in DS neurodevelopment and neurodegeneration, iPSCs derived from different sources have been developed over the years 92. In 2008, Park et al. used four reprogramming factors (OCT4, SOX2, KLF4, and c-MYC) and the retroviruses pMXs to induce fibroblasts from two young male into iPSCs 94. They analyzed the karyotype of these iPSCs and identified the morphology, pluripotency, and differentiation ability of the iPSCs. In 2012, the researchers reprogrammed the iPSCs with fibroblasts from DS patients using lentivirus vectors 95. In an international collaborative investigation, scientists at University College London and the Francis Crick Institute found that patients with Down syndrome carried genes for early-onset Alzheimer's disease (AD) 96. About two-thirds of DS patients have early-onset AD. Therefore, researchers have studied the pathological development of AD, taking advantage of cortical neurons derived from iPSCs with DS 97. They differentiated iPSCs from patients with DS into cortical neurons. Within a few months of culture, these neurons developed the pathology of AD. The pathological marker of AD-hyperphosphorylated tau protein was found to be located on the cell body and dendrites of iPSCs-derived cortical neurons in DS patients, reproducing the later stages AD pathogenesis 97. In 2013, Weick et al. differentiated iPSCs from two DS patients with fibroblasts into neurons. These DS-iPSCs-derived neurons exhibited significant synaptic defects 98. Interestingly, Lu's group produced human DS iPSCs lines from DS mid-pregnancy amniotic fluid (AF) cells through lentiviral transmission and the Yamanaka factor's joint expression. It was found that T21 iPS-NPCs showed developmental defects during neurogenesis. The results showed that T21 AF-iPS cells were a good source for further clarification of DS-induced neurogenesis 99. In 2017, Costa's research team obtained iPSCs from the urinary epithelial cells of 10 DS patients. This technique overcame the previous ethical issues of obtaining stem cells through a skin biopsy. IPSCs produced from urine are also more stable than those obtained from skin biopsies, making them an excellent model for studying DS 100.

Astrocytes play a key role in the pathogenesis of DS. Studies have confirmed that astrocytes derived from DS-iPSCs exhibit cell dysfunction and oxidative stress 101. They interact negatively with iPSCs-derived neurons in neuronal neurite outgrowth, ion channel maturation, synaptic activity formation, and non-cell-autonomous toxic effects. More importantly, the research transplanted DS-iPSCs-derived astrocytes into newborn mice's brains and found that the mice represented impaired brain function 101. The study also showed that DS astrocytes do not promote neurogenesis of endogenous neural stem cells *in vivo*. The antibiotic drug minocycline, which was approved by FDA specifically, corrected the pathological phenotype in DS astrocytes by regulating the expression of S100B, GFAP, inducible nitric oxide synthase, and thrombospondin 1 and 2 101. By combining calcium imaging with genetic approaches, other scholars discovered the functional defects of DS astroglia and their effects on neuronal excitability 102. Compared with the isogenic astrocytes in the control group, DS astrocytes showed more frequent spontaneous calcium ion fluctuations, reducing co-cultured neurons' excitability. Also, the researchers eliminated the spontaneous calcium activity of astrocytes. They saved the inhibited neuronal activity by blocking adenosine-mediated signaling, which knocked out the inositol triphosphate (IP3) receptor or the calcium-binding protein S100B encoded on HSA21 102. This study suggested that DS changed the mechanism of astrocyte function and interfered with the excitability of neurons. Last year, using iPSCs from DS, Bruno HS et al. studied astrocytes' role in DS and the astrocyte secretomeastrocyte secretome's impact's impact in neuronal mTOR signaling and synapse formation 103. Utilizing the DS neurons from hiPSCs, they observed that the Akt/mTOR axis' excessive activation in the DS brain and DS astrocytes might play a key role in this dysfunction. This study demonstrated that 21 trisomy in astrocytes did not only lead to neuronal abnormalities but also influenced the cell-autonomous dysfunctions, which was caused by 21 trisomy in neurons. This research indicated that using iPSCs to study the neuropathogenesis of DS could improve our understanding of DS and contribute to the development of new treatment methods 103.

*In vitro* studies did not fully simulate the pathological process of DS. Therefore, one group transplanted early differentiated hiPSCs-derived neurons into adult mice's cerebral cortex to study human neurons' dynamics* in vivo* 93. Human neurons increased, developed, and differentiated after successfully being transplanted into mouse brains and gradually formed vascularized complex cell structures containing neurons and glia. This allowed researchers to monitor human neurons' development in the physiological microenvironment provided by the mouse brain. Besides, overproduction of astrocytes was detected in the transplant compared to controls, dendritic spinal density and spinal stability increased as well, but both synapse and overall activity reduced 104. A recent study took advantage of iPSCs from DS fibroblasts samples, which showed that DS brain organoids spontaneously developed into pathological signs of AD, such as amyloid plaques and tau pathological components 105. Since DS included two aspects of neurodevelopment and neurodegeneration, organoids may open up new ways to study DS's different cell phenotypes.

## Application of iPSCs in Epilepsy

In addition to the above diseases, Epilepsy is also a kind of neurodevelopmental disorders. In children with epileptic encephalopathy, the gene mutation is an important cause. Related neurodevelopmental HCN1, FGF12, SLC12A5, ARX, PCDH19, KCNA2, DENND5A, AARS, SCN8A, CDKL5, STXBP1, SCN1B, SLC1A2, PLCB1, DOCK7, TBC1D24, SCN2A, SPTAN1, SZT2, GABRB3, KCNQ2, GNAO1 and other genes can all lead to epileptic encephalopathy 106, 107. Epilepsy caused by single-gene mutations is the dominating reason for a variety of epilepsy syndromes 108. Utilizing iPSCs models for genetic epilepsies (GE), gene mutations induced GE by altering the balance between neuronal excitation and inhibition, which is associated, among other factors, with neuronal developmental disturbances, ion channel abnormalities, and synaptic dysfunction 83, 84, 109, 110. Research results from patient-derived iPSCs-derived models also indicated that the increased sodium ions in inhibitory and excitatory neurons were associated with an increased evoked action potential (AP) and spontaneous discharge 111-113. The mutation of the SCN1A gene encoding the alpha subunit of the Nav1.1 channel leads to Epilepsy with various clinical phenotypes 114-116. In the brain, Nav1.1 is mainly expressed in GABAergic neurons and a small number of excitable vertebral neurons 114-116. In 2013, Higurashi et al. 111 firstly generated iPSCs by skin fibroblasts obtained from a Dravet syndrome patient with SCN1A mutation. They continued to generate neurons that primarily were GABAergic which were displayed significantly impaired by using current-clamp analyses confirming that loss-of-function in GABA neuron inhibition was involved in epilepsy Onset. In a study, researchers applied CRISPR/Cas9 and TALEN-mediated gene-editing techniques to iPSCs-based disease models to explore the pathogenesis of Epilepsy caused by SCN1A functional deletion mutations 117. Using CRISPR/Cas9 to fluorescently label GABAergic neuron subtypes in iPSCs-derived neurons, the researchers first conducted electrophysiological studies on nerve subtypes expressing SCN1A and monitored inhibitory and excitatory types of nerves postsynaptic activity. They found that the mutation c.a5768G resulted in the absence of Nav1.1 current in the exogenous transfection system, which affected the nature of the Nav current amount and affected the Nav activation of GABAergic neurons expressing Nav1.1. Two changes in Nav further reduced the amplitude of patient-derived GABA neurons and enhanced these neurons' action potential threshold. Eventually, the spontaneous inhibitory postsynaptic currents (sIPSCs) in the patient-derived neuronal network were reduced. Although spontaneous excitatory postsynaptic currents (sEPSCs) did not change significantly, it was found that the entire postsynaptic activity changed from a state of inhibition to the excitation of a patient-derived neuronal network after further analysis of the frequency of sIPSCs and sEPSCs. This indicated that changes in sIPSCs alone were sufficient to significantly reverse the excitability level of spontaneous post-synaptic activity. These findings revealed the fundamental physiological mechanism of Epilepsy caused by SCN1A loss-of-function mutations 117. Simultaneously, the iPSCs model research confirmed that astrocyte activation 118-120, mitochondrial dysfunction 121, 122, and abnormal signaling pathway activity 123, 124 were also important factors to the molecular mechanisms of GE 108.

In addition to pathogenesis research, iPSCs have also been applied to research on epilepsy treatment 119, 124, 125. DS-iPSCs-derived GABAergic neurons were used to test the efficacy and therapeutic effect of the newly developed drug cannabidiol (CBD). The results showed that these cells were suitable models for testing the drug's mechanism toxicity 125. In one study, human-derived iPSCs of GABA progenitor cells were transplanted into the hippocampus of the early animal model of temporal lobe epilepsy. The results showed that the frequency of seizures was decreased, the loss of GABA-ergic neurons was reduced, and cognitive and emotional functions were also improved 126. Further testing showed that the transplanted human neurons formed synapses or connections with the host's excitatory neurons, and their GABA and other inhibitory interneuron special markers were positive 126. Another intriguing finding was that the implanted human GABAergic neurons seem to be directly involved in controlling seizures, so silencing these neurons led to an increase in the number of seizures 126. This discovery was of great significance for treating this incurable brain disease, which showed a potential method and inspired significance for other diseases such as Parkinson's and Alzheimer's.

## Application of iPSCs in other neurodevelopmental disorders

In addition to the three neurodevelopmental disorders summarized above, there are many other neurodevelopmental disorders, such as Microcephaly, Tourette Disorder (TD), and so on.

In 2013, researchers used RNA interference technology and patient-specific iPSCs to establish an organoid microcephaly model 127, mimicking the temporal development of neuronal subtypes layering of tissue. In the “Proof of Principle” experiment, the authors employed induced pluripotent stem cells from patients to generate a “microcephaly” model describing neural differentiation defects not previously observed in rodent models 127. Compared with the control group, the patient-derived organoids showed a smaller neuroepithelium area. The overall size was smaller, which simulated the most typical microcephaly symptoms. The brain organoids formed by hiPSCs mimicked human embryos' development, so it was used to diagnose whether pregnant women were infected with the Zika virus and how it caused Microcephaly in infants 128. Qian X et al. have used iPSCs to induce the generation of brain organoids imitating the 3D tissues of developing organs 129. When infecting the brain organoids with the Zika virus, they discovered that the Zika virus first attacked neural stem cells rather than new neurons. Pathogens resulted in increased death of neural stem cells and creased the number of neurons in the cortex, eventually leading to Microcephaly. Based on this research, there have been many reports on the use of brain organoids to explore Microcephaly's mechanism. These reports confirmed that ZIKV infection caused an overall decrease in the size of organoids. ZIKV also induced apoptosis in neural precursor cells, weakens the proliferation of precursor cells, and increased the size of the ventricular structure's inner cavity 130-132. Congenital Microcephaly, also known as true MMicrocephaly or Autosomal Recessive Primary Microcephaly (MCPH), is a neurological development disorder 133, 134. Its clinical feature is a reduction in head circumference with a certain degree of non-progressive intellectual degradation. The causes of Microcephaly include environmental factors, genetic factors, and infection factors. At present, it is generally believed that MCPH is multi-gene stealth inherited disease 133, 134. One team differentiated the iPSCs of patients with autosomal recessive Microcephaly caused by CDK5RAP2 gene mutation into brain-like tissues and found that the patients showed defects in the size and division of neural progenitor cells, abnormalities in the direction of the central axis, and premature Neurotic division. These abnormalities could be partially restored by overexpressing the wild-type CDK5RAP2 gene 127.

Tourette Syndrome (TS) is a chronic disease with a neurobiological basis in the brain's basal ganglia in children. It also is a childhood-onset neuropsychiatric and neurodevelopmental disorder characterized by the presence of both motor and vocal tics 135. One group characterized the functional consequences of the mutation in iPSCs-derived neurons and demonstrated the self-oligomerization of the PNKD (L) protein and its interaction with the synaptic active zone protein RIMS1α. This report enhanced the evidence that PNKD played a critical role in the neurodevelopment and function of iPSCs 136. Therefore, we hope to use iPSCs to study TS-related pathogenesis and explore better ways to prevent and cure the disease.

As well as, iPSCs lines of some other rare children's neurological disorders, including Duchenne muscular dystrophy 137, Chubby Willy syndrome 138, and Mucopolysaccharidosis IIIB (MPSIIIB) 139 have been established. But whether these cells truly represent the pathogenesis and phenotype of these diseases needs further research.

## IPSCs clinical application challenges

IPSCS has similar pluripotency characteristics as ESCS but possesses an unlimited source and less ethical issues, as well as it has advantages in immune tolerance and personalized intervention. The disease models from the cellular-2D level to the organoid-3D level constructed with iPSCs solve the inadequacies of the simulation of nervous system models and the difficulty of obtaining human body materials and meet the needs of studying the pathogenesis of neurodevelopmental disorder in children. However, low generation efficiency, genetic instability, epigenetic abnormalities, and tumorigenicity may all be the difficulties faced by iPSCs technology 140. Furthermore, the cost of establishing clinical-grade iPSCs for each specific patient is tarnal high and time-consuming 141, 142. Whether the pluripotency and differentiation potential of iPSCs is the same as or at least equivalent to ESCs is still worth being explored as well.

## Tumorigenicity

The promotion of disease models based on iPSCs derived from specific patients shows the great potential of utilizing this technology in regenerative medicine. However, the transformation of this technology into the clinical treatment stage requires many preclinical experiments to evaluate its safety and effectiveness, which is necessary. Unlike ESCs, human iPSCs are highly similar in marker expression, self-renewal ability, and differentiation potential. However, iPSCs cannot be equated to ESCs. Genome-wide genetic and epigenetic studies of iPSCs have shown that their genes are unstable, epigenetic memory persists and varying genetic variation degrees 143. The six inducing factors (Oct3/4, Sox2, c-Myc, Klf4, Lin28, Nanog) used in iPSCs technology, almost all are oncogenes, whose overexpression were often associated with tumors. Only Lin28 is not related to tumorigenesis 144. The tumorigenic potential of embryonic stem cells was dose-dependent. When Oct4 was overexpressed, tissues, or organs derived from the embryonic stem cells were highly malignant, while the inactivation of Oct4 reduced the malignant potential 145. Besides, Sox2 is an oncogene in lung and esophageal squamous cell carcinoma 146. As well as C-Myc was a proto-oncogene detected in a variety of tumors 147. It was found that the lack of c-Myc in the combination of Oct4, Sox2, and Klf4 could lead to iPSCs induction failure 148. Its tumorigenic properties inhibited reprogramming and also increased the frequency of cell turnover during the passage of iPSCs. C-Myc transgene in iPSCs sustained effects increasing the risk of tumor formation 148. Whether klf4 is a tumor suppressor gene or an oncogene is determined by the environment. While Nanog promoted the occurrence and metastasis of breast cancer 149. Moreover, conventional methods concerning reprogramming iPSCs possessing functions similar to embryonic stem cells require multiple viral transduction factors. It was found that each iPSCs clone contained 3-6 retrovirus sets, each clone had more than 20 retrovirus binding sites, and each site might increase the risk of cancer 150. Overexpression of oncogenes may make cells grow out of control or even become cancerous. When used as a recombination factor, they can change the recombined cells 151. The first generation of iPSCs was obtained by retroviral transfection with specific transcription factors. The genetic material inserted through the retroviral vector was randomly integrated into the host genome, which caused genetic abnormalities and teratoma formation 152. The researchers used bioinformatics tools to analyze all available data of the iPSCs cell lines constituted by 11 different cells' reprogramming. They found that 593 shared genes in iPSCs, 209 of which were in human tumor cell lines and tumor tissues. Moreover, five oncogenes were overexpressed in these iPSCs, which indicated that the expression of common genes in iPSCs has a certain risk of tumor and cancer 153. To explore the tumorigenic tendency of human iPSCs, scientists studied the gene expression and DNA methylation of 49 human iPSCs cell lines and ten human ESCs cell lines. It was found that seven iPSCs clones retained many undifferentiated cells and even formed teratomas after neuronal differentiation culture and transplantation into rat brain. These defective differentiated cell lines showed a high gene expression level for several genes, including human endogenous retrovirus long terminal repeats, hiPSCs abnormal gene expression and defect potential during neural differentiation 154. Subsequent iPSCs dismissed virus integration and engaged PiggyBal transposon, adenovirus or plasmid transfection, direct reprogramming protein, small molecule induction, etc. to obtain iPSCs without any DNA manipulation. The method for reprogramming can generate iPSCs without the integration of foreign DNA into the host genome. Therefore, the risk of tumor development by integrating viruses or reprogramming factors into the genome is avoided by non-integration technology. Moreover, there are also mutations that occur during cell induction. Some somatic cells genes were damaged during the induction process, and the induction itself also leads to increased mutation copy. And the longer the cultivation time, the more the accumulation of variation 155, 156. In addition, different detection methods provide different results for the genetic stability of induced pluripotent stem cells. Modern molecular genetic analysis techniques have found more genetic abnormalities in induced pluripotent stem cells rather than traditional cytogenetic analysis techniques.

## Transplant immunogenicity and safety

Although it is generally believed that iPSCs produced from autologous cells might avoid immune rejection, the redifferentiation mechanism of iPSCs after transplantation into the body is not clear, and the autologous transplantation of iPSCs may behave immunogenic 48. It was recently reported that there were immunogenic differences between ESCs and iPSCs differentiated endoderm 157. There is currently no specific detection system to evaluate functional cells' efficiency and safety after transplantation. The feeder layer cells used for the initial iPSCs culture* in vitro* were mouse embryonic fibroblasts, which required the addition of necessary growth factors or the removal of inhibitory factors to achieve cell self-renewal. But it is worth noting the safety issues of animal-derived serum, including the possible presence of immunologically active substances, animal viruses, and infectious proteins. There may be some difficult components to control. Therefore, the subsequent studies mostly use the serum-free complete medium to avoid the above safety hazards. It can be used to induce and maintain iPSCs and provide growth factors and nutrients necessary to support iPSCs self-renewal and maintain pluripotency.

## Low induction efficiency and high cost

Although there have been breakthroughs in the technology of iPSCs reprogramming, the low induction efficiency is a major obstacle that must be overcome in the current clinical application of iPSCs. Although the efficiency of iPSCs colony formation varies with different donors, the endogenous expression of basic factors is positively correlated with cell reprogramming efficiency. The average efficiency of iPSCs clone formation from donor cells with basic endogenous factor expression was 0.49±0.10%, the general transduction efficiency was 0.31-0.66%, the average transduction efficiency of neonatal skin fibroblasts is 0.03%, and the average efficiency of iPSCs cloning was 0.02~0.03%^158^. Also, when multiple samples need to be reprogrammed, the high cost derived from iPSCs is another factor limiting most laboratories' development. Moreover, among the many widely used non-integration methods, the Sendai virus and mRNA method require expensive reagents for reprogramming. In contrast, the episomal method requires a large number of starting cells and high labor costs.

Moreover, most iPSCs disease models currently used 2D models whilst 3D organoid technology is still in its infancy. Since the interaction between different types of cells may also play a key role in disease occurrence, these models may not reveal the complexity of the disease pathology entirely 159. To achieve human disease tissue repair and organ regeneration, stem cell research also depends on the cross-fusion and breakthrough of multidisciplinary technologies such as medicine, life sciences, engineering, and materials science. This technology's clinical transformation and industrialization still face numerous challenges, such as high cost, tumorigenicity, low induction efficiency, and limited disease phenotype.

Although the problems related to the clinical application of iPSCs need to be further resolved, iPSCs technology still represents an outstanding achievement on the neurodevelopmental disorder in children. It is engaged in the discovery and toxicity testing of drugs for neurodevelopmental disorders and has an application in neurodevelopmental disorder models, nerve cell transplantation, and clinical trials. It has meaningful significance for future medical research on neurodevelopmental disorders. IPSCs will also become a crucial tool for brain-like organs or the pathogenic mechanism of neurodevelopmental disorders. It is a promising source of neural progenitor cells based on cell therapy development in regenerative medicine. Besides, iPSCs can be genome-edited by homologous recombination to understand the mechanical relationship between the patient's genotype and cell phenotype. This feature further enhances the potential application of iPSCs from basic research to regenerative medicine. Shortly, this innovative technology will make more progress and become an indispensable tool in future biomedical research on neurodevelopmental disorders. With the emergence of new technologies, new methods, and new disease models, iPSCs technology would facilitate clinical disease research. IPSCs technology is expected to promote further the clinical practice of precision medicine in clinical diseases.

In short, the application of any new technology will go through a long process, and iPSCs technology is no exception. Making better use of new technologies of stem cells and interdisciplinary services to serve humanity will be a new topic that we need to study more from now on.

## Abbreviations

iPSCs: induced pluripotent stem cells
ESCs: embryonic stem cells
GDD/ID: generalized stunting/mental disability
ASD: autism spectrum disease
ADHD: attention deficit hyperactivity disorder
hiPSCs: human induced pluripotent stem cells
hESCs: human embryonic stem cells
NDDs: neurodevelopmental disorders
CNSDs: central nervous system diseases
HTS: high-throughput screening
RTT: Rett syndrome
FXS: Fragile X syndrome
LTCs: L-type voltage-gated calcium [1]channel
REST: RE-1 silent transcription factor
miRNA: microRNA
DS: Down Syndrome
AD: Alzheimer's disease
AF: amniotic fluid
IP3: inositol triphosphate
GE: genetic epilepsies
AP: action potential
sIPSCs): spontaneous inhibitory postsynaptic currents
sEPSCs: spontaneous excitatory postsynaptic currents
CBD: cannabidiol
TS: Tourette Syndrome
HexA: hexosaminidase A
HexB: hexosaminidase B
MPSIIIB: Mucopolysaccharidosis IIIB
